# Genome Characterization of Temperate Bacteriophages and Associated Genetic Features in Avian Pathogenic *Escherichia coli* from Brazilian Poultry

**DOI:** 10.3390/ani16081159

**Published:** 2026-04-10

**Authors:** Rafael Dorighello Cadamuro, Giulia Von Tönnemann Pilati, Mariana Alves Elois, Álvaro Cañete Reyes, David Rodríguez-Lázaro, Gislaine Fongaro

**Affiliations:** 1 Department of Microbiology, Immunology and Parasitology, Federal University of Santa Catarina, Florianópolis 88040-900, SC, Brazil; giuliavpilati@gmail.com (G.V.T.P.); maarianaeloiss@gmail.com (M.A.E.); gislainefongaro@gmail.com (G.F.); 2 Microbiology Division, Faculty of Sciences, University of Burgos, 09001 Burgos, Spain; alvarocanetereyesg@gmail.com; 3 Research Centre for Emerging Pathogens and Global Health, University of Burgos, 09001 Burgos, Spain

**Keywords:** avian pathogenic *Escherichia coli* (APEC), phageome, whole-genome sequencing, phage diversity

## Abstract

To characterize the ecological and genomic architecture of temperate bacteriophages in *Escherichia coli* isolated from Brazilian broiler chickens, we analyzed 63 femur-derived genomes, most fulfilling molecular avian pathogenic *E. coli* (APEC) criteria, and tested whether temperate phage regions are enriched for antimicrobial resistance genes (ARGs), virulence factors, plasmid markers, and other mobilome components. Diversity was summarized using incidence-based richness estimators and bootstrap confidence intervals, and positional enrichment was assessed using permutation-based statistical analysis. We detected 1164 phage-like elements, including 188 medium- and high-quality phages, of which 93.6% were temperate. Median temperate diversity per genome was three phage genera and three temperate regions. At the population level, 19 temperate genera were observed, with a Chao2 estimate of 21.2, indicating near-saturated genus-level diversity. Positional mobilome analysis showed significant enrichment of insertion sequences within temperate regions (*p* < 0.05), while ARGs, virulence factors, and plasmid markers were not significantly enriched inside temperate phage coordinates (*p* > 0.05). The surrounding genomic neighborhood (±20 kb) accumulated mobile elements but showed no significant enrichment. CRISPR spacer matches further supported ongoing host–phage interactions. Overall, temperate phages are widespread and ecologically structured in Brazilian broiler-associated *E. coli*, but they are not preferential hotspots for ARG, virulence, or plasmid gene enrichment; instead, they are chiefly associated with insertion-sequence enrichment.

## Simple Summary

Temperate bacteriophages are viruses that infect bacteria and can integrate into the bacterial genome, potentially influencing bacterial evolution and the spread of antibiotic resistance or virulence. Poultry-associated *Escherichia coli* can cause disease in birds and may also pose risks to food safety and public health. In this study, we analyzed genomes of *E. coli* isolated from Brazilian broiler chicken femurs to understand the diversity and ecological role of temperate bacteriophages and to determine whether these viral regions concentrate genes linked to antibiotic resistance, virulence, plasmids, or other mobile genetic elements. We found that temperate bacteriophages were widespread and associated with certain conserved taxa, with most bacterial genomes carrying multiple viral regions. However, these regions were not preferential hotspots for antibiotic resistance or virulence genes. Instead, they were enriched in insertion sequences, small mobile DNA elements that promote genetic rearrangements. Evidence from bacterial immune memory systems indicated ongoing interactions between bacteria and viruses. These findings improve our understanding of how viruses shape bacterial genomes in food-production animals.

## 1. Introduction

The poultry sector represents one of the most important animal protein production systems worldwide. According to the latest data from the United States Department of Agriculture (USDA), global chicken meat production reached approximately 103.8 million metric tons during the 2023/2024 marketing year, ranking as the second-largest meat production sector globally [1]. Production is highly concentrated in a limited number of regions, with the United States (20%), China (15%), Brazil (14%), and the European Union (11%) collectively accounting for approximately 60% of total global chicken meat production [1].

Although *Escherichia coli* is a common member of the intestinal microbiota of healthy birds, a subset of strains exhibits enhanced pathogenic potential and is associated with systemic and respiratory infections, particularly under environmental and management-related stress conditions [2,3]. The control of avian colibacillosis relies primarily on antibiotic therapy combined with biosecurity and sanitation measures; however, the extensive use of antimicrobial agents in poultry production exerts strong selective pressure, promoting the emergence and dissemination of antibiotic-resistant *E. coli* strains [4,5,6].

In recent years, the increasing prevalence of extended-spectrum β-lactamase (ESBL)-producing *E. coli* and strains carrying plasmid-mediated AmpC β-lactamases of avian origin has raised major concerns due to the potential transmission of these strains to humans through the food chain [7,8]. While plasmids and other mobile genetic elements are well recognized as key drivers of antimicrobial resistance gene dissemination, increasing attention has been directed toward bacteriophages, particularly temperate bacteriophages, as additional contributors to horizontal gene transfer [9]. Through mechanisms such as transduction and lysogenic conversion, bacteriophages may facilitate gene mobilization and promote bacterial adaptation under selective pressures [10]. Although the extent of their contribution to the spread of ARGs in natural and agricultural environments remains under debate, the high abundance of bacteriophages in bacterial ecosystems suggests that they may represent a relevant and still underexplored pathway for gene dissemination in intensive poultry production systems [11,12,13,14].

The present study aimed to investigate the ecological dynamics of bacteriophages, antimicrobial resistance genes, virulence factors, mobile genetic elements, and bacterial defense systems within *E. coli* isolates obtained from the femurs of Brazilian broilers (*Gallus gallus domesticus*), the majority of which fulfilled the molecular criteria for APEC. Because bone marrow is typically a sterile anatomical site, the recovery of *E. coli* from this compartment is indicative of systemic dissemination and high invasive potential, providing a biologically relevant framework for exploring phage–host interactions in highly pathogenic strains circulating in poultry production systems [15,16].

Using whole-genome sequencing (WGS), we explored associations between bacteriophage populations and bacterial genomic features, including antimicrobial resistance determinants, virulence factors (VFs), plasmid-related elements, and bacterial defense systems. This approach expands current knowledge on the prevalence and ecological roles of temperate bacteriophages and their potential epidemiological relevance. This study uses assembled genomes from previous research to investigate, for the first time, the dynamics of phages in temperate *E. coli* strains derived from bird femurs to investigate the ecological organization of temperate phages and their association with mobilome and bacterial defense features.

## 2. Materials and Methods

### 2.1. Data Collection

The bacterial genomes analyzed in this study were obtained from a previously published collection of femur-derived *E. coli* isolates recovered from broiler chickens and described by Pilati et al. (2024) [15]. The dataset comprises 63 isolates collected between August and November 2021 from poultry batches originating from six major poultry-producing Brazilian states (Paraná, Santa Catarina, Rio Grande do Sul, São Paulo, Minas Gerais, and Ceará). In the original study, isolates were classified as avian pathogenic *E. coli* (APEC) when they carried at least three of the five minimal virulence predictors *iroN*, *ompT*, *hlyF*, *iss*, and *iutA*, as determined by conventional PCR.

According to the original study, the necropsied broilers were between 13 and 32 days old and presented respiratory clinical signs, including sneezing, rattling, and nasal discharge. Isolates were obtained from femur samples, which are commonly associated with systemic infection and invasive *E. coli* strains. In the original study, bacterial isolates were preserved in Luria–Bertani broth supplemented with glycerol (5:1) and stored at −80 °C prior to genomic DNA extraction. Genomic DNA was extracted using an adapted phenol-chloroform protocol, and sequencing libraries were prepared with the Illumina DNA Prep-Nextera kit and quantified with the Collibri Library Quantification Kit. Whole-genome sequencing was then performed on an Illumina NextSeq platform using paired-end 2 × 150 bp reads (Illumina, San Diego, CA, USA). According to the original study, reads with Phred scores below 20, as well as adapter and low-quality segments, were removed before assembly. Genome assembly was performed using oneshotWGS v1.9, which incorporates the A5 workflow, assembly statistics were obtained with QUAST v5.2.0, and genome annotation was performed with Prokka v1.14.6 [17,18].

Genome assemblies generated in the original study were deposited in NCBI under BioProject PRJNA917297 and were retrieved for this study. Here, the deposited assemblies were reused to characterize temperate phage elements, mobilome components, and bacterial defense systems, without de novo genome reassembly, additional contig-level filtering, or bacterial genome reannotation. Assembly-level quality metrics for the bacterial genomes, including genome size, contig count, N50, GC content, sequencing coverage, and CheckM-based completeness and contamination estimates, were obtained from the publicly available NCBI assembly records associated with BioProject PRJNA917297 and are summarized in Supplementary Table S1. Because the present study focused on phage-associated regions, viral quality curation with CheckV represented the main quality-control step in the current reanalysis, and inferential analyses were restricted to medium- and high-quality phage sequences.

### 2.2. Bioinformatic Analysis

Antimicrobial resistance genes (ARGs), virulence factors, and plasmids were identified using ABRicate [19], screening genome sequences against the ResFinder [20], CARD [21], Megares [22] and Virulence Factors Database (VFDB) with coverage and identity thresholds of 90% [23]. To provide genomic context for phage ecology analyses, genome counts of ARGs, VFs, and plasmid replicons were calculated for each bacterial genome, and deduplication was based on contig identity and feature start/end coordinates.

Plasmid-associated markers were included to assess whether temperate phage regions also contain signatures of other mobile replicative modules, which may reflect mosaic mobilome organization or phage–plasmid-like architectures rather than complete plasmid structures.

Phage identification within genomes was conducted using VIBRANT software (Virus Identification By iteRative ANnoTation), version 1.2.1 [24], followed by quality assessment with CheckV v1.0.3 [25]. Only phage contigs classified as medium or high quality by CheckV were retained for downstream analyses, corresponding to ≥50% completeness and ≤10% contamination for medium-quality sequences and ≥90% completeness and ≤10% contamination for high-quality sequences. A total of 1164 phage-like elements were filtered and 188 remained after CheckV curation, of which 176 were predicted to have a temperate lifestyle and 31 were classified as integrated prophages. Because genome assemblies were generated exclusively from short-read Illumina data, direct evidence of chromosomal integration could only be confirmed for a subset of temperate phage sequences. Throughout this study, the term prophage is reserved for CheckV-classified provirus/integrated elements, whereas other viral regions with predicted lysogenic potential are referred to as temperate phage regions or predicted temperate phage sequences. Accordingly, analyses distinguishing integration status were interpreted conservatively: the term prophage was applied only to CheckV-supported integrated/provirus elements, whereas the broader temperate fraction was discussed as temperate phage regions.

Defense-related systems were annotated using DefenseFinder v2.0.1 [26], whereas integrons were detected with IntegronFinder v2.0 [27]. Insertion sequences and transposases were identified using ISEScan v1.7.3 [28], tRNA genes were annotated with tRNAscan-SE v2.0.12 [29], and MobileElementFinder v1.1.2 [30] was used to characterize mobile genetic elements (MGEs) across genomes and Mob-suite v3.1.9 [31]. CRISPR (Clustered Regularly Interspaced Short Palindromic Repeats) arrays and CRISPR–Cas systems were identified using CRISPRCasFinder v 4.3.2 [32], and CRISPR spacer sequences were extracted for downstream comparative analyses using SpacePHARER Release 5-c2e680a Latest 21 April, 2021 [33]. Phage taxonomy was assigned using three complementary tools, BLAST+ 2.17.0, PhageGCN implemented in PhaBOX 2.1.13, and Phagenus [34]. BLASTn classification was based on best-hit alignment metrics, including sequence identity and query coverage, using the RefSeq Viral database (updated 20 November 2025).

Blastn was applied using a maximum E-value threshold of 1 × 10^−20^, a minimum nucleotide identity of 85%, and a minimum alignment length of ≥500 bp or ≥5% of the query contig length (whichever was greater), retaining up to 50 target sequences per query and summarizing taxonomy by a top-10 bitscore-ranked hit consensus requiring ≥3 valid hits and ≥60% majority support. PhageGCN uses a graph convolutional network to infer viral taxonomy based on shared genomic features [35]. Final taxonomic assignment was based on concordant classification among tools when available, or on recovery by at least one tool when concordance could not be established. Phage lifestyle was predicted using the PhaTYP module implemented in the PhaBOX2 framework, which classifies viral sequences as temperate or virulent [36]. Taxonomic interpretation followed the current ICTV framework, acknowledging the substantial reclassification introduced from 2022, which resulted in many lineages being reassigned as unclassified at the genus or family level [37].

Phages were taxonomically classified according to the current viral taxonomy, noting that significant reclassification occurred in 2022 due to polyphyletic group reorganizations. Consequently, viruses lacking clear evolutionary lineage or defining features lost specific genus or family classifications and were reassigned to an ‘unclassified’ status, accompanied by their respective class names [38].

### 2.3. Statistical Analysis

All statistical analyses and figure generation were performed in Python 3.14.3 using pandas 3.0.1, numpy 2.4.3, scipy 1.17.1, statsmodels 0.14.6, matplotlib 3.10.8, and seaborn 0.13.2. Analyses included bootstrap confidence intervals, incidence-based Chao2 richness estimation, geometric permutation tests, Wilson confidence intervals, and Spearman rank correlation. Statistical significance was defined as *p* < 0.05, and multiple comparisons were corrected using the Benjamini–Hochberg false-discovery-rate procedure, with adjusted values below 0.05 considered significant where applicable.

#### 2.3.1. Richness Diversity of the Temperate Phage Genus Pool

Temperate phage diversity was quantified at the genus level using incidence-based metrics. At the genome level, we summarized (i) temperate genus richness per genome (temperate_richness) and (ii) the number of temperate phage regions per genome (n_temperate). Because these distributions were asymmetric, summary statistics are reported as medians with 95% confidence intervals estimated by bootstrap resampling (1000 resamples). To describe gamma diversity across the full temperate phage genus pool, we constructed a genome-by-genus incidence matrix (presence/absence of each genus across genomes) using genera assigned to temperate phage records. Total genus richness was estimated using the incidence-based Chao2 estimator. Sampling completeness was evaluated using sample-based accumulation curves generated by random permutations of genome order (200 permutations), reporting the median curve and its 95% permutation band. Curve saturation was summarized by the terminal slope computed over the last 10% of genomes (minimum of two points) and by the number of genomes required to reach 90% of the final cumulative richness.

#### 2.3.2. Enrichment of ARG, Virulence, and Plasmid Markers Within Temperate Phages

To assess whether mobilome features concentrate in the genomic context of temperate phages, we defined three positional compartments for each feature (ARG, virulence factor, and plasmid marker) relative to temperate phage coordinates on the same contig: Inside (overlapping the temperate phage region), Flank (located in the genomic neighborhood surrounding the region), or Outside (all remaining positions). The Flank compartment was evaluated at a spatial scale of 1000 bp to capture immediate boundary-associated features, particularly for ARG carriage near the flanks of temperate phage insertion sites. For each temperate phage region, we recorded whether at least one feature from each category occurred inside the phage region or within its flanking genomic neighborhood. To characterize the presence of ARGs, VFs, and plasmids, two subsets were made, one containing all phages and the other containing only phages with medium and high quality. Statistical analyses were conducted only with medium- and high-quality phages, considering the higher precision and accuracy in phage-like elements and in the boundaries of these elements within the genome.

For descriptive visualization of the most recurrent ARGs, virulence factors, and plasmid-associated markers were collapsed into broader functional or family-level categories to reduce fragmentation and facilitate interpretation of the dominant patterns across phage-associated compartments.

Statistical support for enrichment was obtained using a geometric permutation test (1000 permutations per temperate phage region). Specifically, each temperate phage region was randomly repositioned along its contig while preserving its length, and the Inside/Flank feature assignments were recalculated to generate a null distribution of counts. Empirical *p*-values were computed as the fraction of permutations with counts ≥ the observed value (with a +1 correction), and multiple testing was controlled by Benjamini–Hochberg FDR. Empirical *p*-values < 0.05 were considered significant, and results were reported as significant only if they remained below FDR < 0.05 after Benjamini–Hochberg correction.

#### 2.3.3. Mobilome Architecture Around Temperate Phages

Two spatial scales were considered in positional analyses: an immediate flanking interval (±1 kb) to capture local boundary-associated features adjacent to temperate phage regions, and an extended neighborhood (±20 kb) to evaluate broader mobilome accumulation patterns surrounding these regions.

A complementary analysis characterized the broader mobilome landscape associated with temperate phages, including insertion sequences and transposons, integron components (integrases and associated elements), tRNAs, and plasmid mobility markers (replicons, relaxases, oriT, and MPF systems). Using the same compartment definition (Inside, Flank, Outside) and flank sizes (1000 bp), mobilome features were mapped relative to temperate phage coordinates on the same contig. As in the previous analysis, we considered two datasets: one including all phages and another including only medium- and high-quality phages.

For each temperate phage region, the occurrence of mobilome elements within these compartments was recorded, enabling descriptive characterization of hotspot-like genomic architectures, such as temperate phage regions collocated with mobile elements and/or tRNAs. Enrichment testing used the same geometric permutation framework described above (1000 permutations per temperate phage region; empirical *p*-values with +1 correction; Benjamini–Hochberg FDR), ensuring consistent statistical inference across mobilome layers and spatial scales. Significant enrichments were defined as those with an FDR-adjusted *p*-value < 0.05.

#### 2.3.4. Integrated Defense Ecology: CRISPR Spacers, Defense Systems, and Phage-Targeted Signals

To integrate bacterial defense capacity with viral pressure, we combined: (i) CRISPR summaries per genome, including spacer counts and Cas presence when detected; (ii) defense system profiles, number and types of defense systems per genome (defense burden); (iii) spacer-to-target alignments. Spacer hits were filtered using minimum identity and alignment coverage thresholds. Downstream analyses focused on targets with evidence consistent with bacteriophage origin, while retaining ambiguous assignments as unclassified to avoid over-interpretation. Taxonomic assignment of CRISPR spacer targets was inferred by mapping SpacePHARER hits to their corresponding NCBI nucleotide accessions and retrieving lineage information through the NCBI Entrez taxonomy database. Only targets consistent with bacteriophage origin were retained for downstream summaries, whereas ambiguous assignments were kept as unclassified.

Defense system prevalence across genomes is reported as proportions with Wilson 95% confidence intervals. Associations between defense burden and CRISPR memory (defined here as the total number of CRISPR spacers per genome) were evaluated using Spearman rank correlation, with 95% bootstrap CIs (2000 resamples). To describe focused targeting, we additionally quantified within-genome redundancy of spacer events against the same phage genus (unique events per genome–genus pair), summarizing the proportion of genomes exhibiting ≥2 independent targeting events to a given genus.

## 3. Results

### 3.1. Genetic and Phage Diversity in E. coli

**Figure 1:**
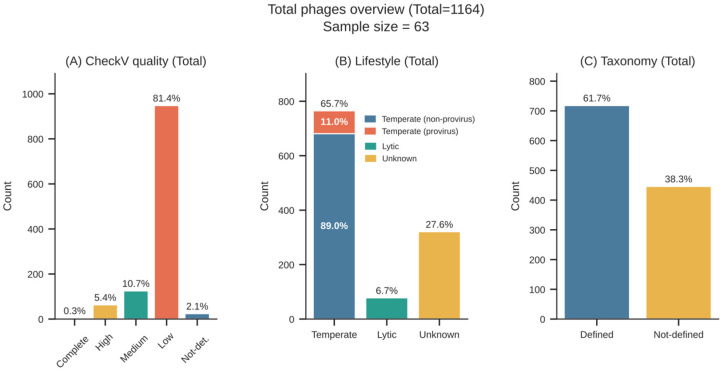
Overview of phage features across the dataset. (**A**) Quality classes of detected phage sequences according to CheckV. (**B**) Lifestyle classification of detected phages, highlighting temperate, virulent, and integrated prophage-associated elements. (**C**) Taxonomic resolution of detected phages, showing the proportion assigned to a defined taxonomic rank of genus or remaining unclassified.

Across the 63 *E. coli* genomes, a total of 1164 phage-like elements were identified. The majority of these elements were classified by CheckV as low quality (81.4%), whereas smaller fractions corresponded to medium- (10.7%) and high-quality (5.4%) sequences, which were retained for downstream ecological and functional analyses, as shown in Figure 1A. The majority of low-quality viral regions are related to genome fragmentation resulting from short-read Illumina sequencing [25]. Lifestyle prediction analyses showed that a fraction of the phage-like elements was undetermined (27.6%), a smaller proportion was classified as lytic (7.6%), and most were predicted to be temperate (65.7%). Among the temperate phages, only 11% were confirmed as integrated by CheckV, as illustrated in Figure 1B. It was possible to determine the viral genus for 57.9% of all detected phages, whereas taxonomy could not be resolved for 42.1% of the sequences, as shown in Figure 1C. The full taxonomic panel is presented in Supplementary Figure S1. In total, only 60 strains presented medium or high-quality phage sequences, comprising 188 phages. Of these, 176 (93.6%) were classified as temperate and 12 (6.4%) as virulent. Among the temperate phages, 31 were confirmed as integrated prophages. These 188 medium- and high-quality phage sequences, including 176 temperate predictions, constituted the high-confidence dataset used for downstream analyses.

**Figure 2:**
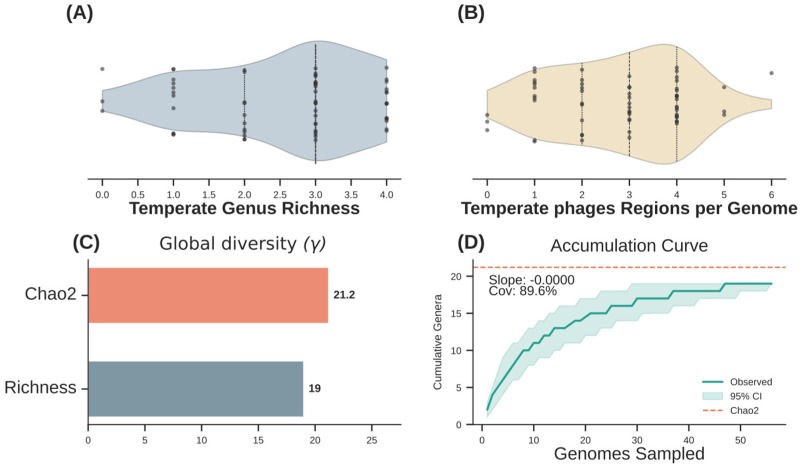
Genome-level and global diversity of temperate phages. (**A**) Temperate phage genus richness per genome. (**B**) Number of temperate phage regions per genome. (**C**) Observed and estimated global genus richness based on the Chao2 estimator. (**D**) Sample-based accumulation curve of temperate phage genera across genomes.

To characterize the diversity of temperate phages across the dataset, we quantified temperate phage diversity per bacterial genome using two complementary metrics. First, we determined the richness of temperate phage genera per bacterial genome, which ranged from 0.0 to 4.0 (Figure 2A). We then assessed the number of temperate phage regions per genome, which varied from genomes without medium- or high-quality phages to genomes harboring up to six temperate phage regions (Figure 2B). Substantial heterogeneity was observed among genomes, both in the number of temperate phage genera detected and in the number of temperate phage regions per host genome, indicating an uneven distribution of temperate phages across the bacterial population. Global diversity analysis revealed an observed richness of 19 genera and a Chao2 estimate of 21.2, as shown in Figure 2C. An accumulation curve based on genus-level phage taxonomy indicated a sampling coverage of 89.6% of the estimated richness (Figure 2D), and 31 genomes were sufficient to recover 90% of the final cumulative genus richness.

### 3.2. Phage-Associated Mobilome Landscape: Compartmentalization and Enrichment Around Temperate Phages

To characterize the mobilomic architecture associated with temperate phages, we first quantified the global pool of mobile genetic elements across the analyzed genomes. In total, 3821 tRNA genes, 1723 mobile elements (including insertion sequences and Miniature Inverted-repeat Transposable Elements—MITEs), 1934 plasmid-associated markers, and 313 integron-related components were detected across the dataset, as well as 176 temperate phage regions.

**Figure 3:**
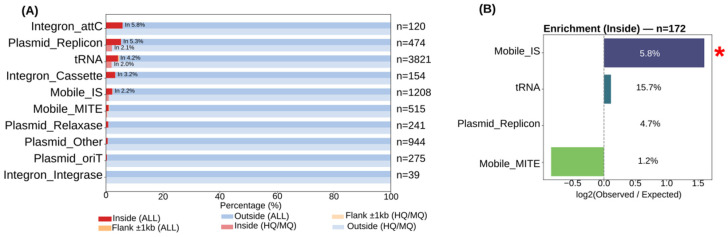
Mobilome context of temperate phage regions. (**A**) Distribution of mobile genetic element categories inside temperate phage regions in the all-phage and medium-/high-quality subsets. (**B**) Enrichment of mobile genetic elements within temperate phage regions relative to a permutation-based null model. The asterisk (*) indicates statistical significance.

Landscape analyses compared features located inside temperate phage regions in two datasets, one including all predicted temperate phage regions and the other restricted to medium- and high-quality temperate phage regions, as well as in their surrounding 20 kb genomic neighborhoods, revealing a spatial pattern (Figure 3A).

Comparing both datasets, mobile genetic elements were predominantly located outside temperate phage regions, MITEs were only rarely observed inside temperate phages (0.97% in All; 0.39% in HQ/MQ), whereas tRNAs showed the highest relative occurrence within phage regions among the analyzed categories (4.24% in All; 1.99% in HQ/MQ).

Insertion sequences and plasmid replicons were also more frequently detected inside temperate phage regions than in flanking regions, with higher proportions observed in the All dataset (insertion sequences: 2.24% inside vs. 97.68% outside; plasmid replicons: 5.27% inside vs. 94.73% outside) and reduced but still detectable proportions in the HQ/MQ subset (insertion sequences: 0.99% inside vs. 99.01% outside; plasmid replicons: 2.11% inside vs. 97.89% outside). All enrichment analyses were restricted to the medium- and high-quality subset. In the medium- and high-quality subset, enrichment analyses further identified insertion sequences as significantly enriched within temperate phage regions (log_2_ observed/expected > 1, FDR < 0.05; Figure 3B), indicating preferential incorporation of these elements into temperate phage genomes. In contrast, analyses of the surrounding genomic environment did not reveal statistically significant enrichment patterns in the flanking regions.

#### 3.2.1. Functional Landscape of Resistance, Virulence, and Plasmid-Associated Genes Surrounding Temperate Phage Predictions

**Figure 4:**
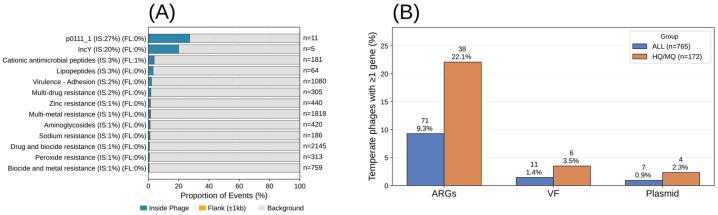
Distribution and enrichment of resistance, virulence, and plasmid-associated genes in temperate phage regions. (**A**) Distribution of ARGs, virulence factors, and plasmid-associated markers across predicted temperate phage regions, immediate flanking regions (1 kb), and the remaining genomic background in the all-temperate-phage dataset and in the medium-/high-quality subset. (**B**) Proportion of temperate phage regions carrying at least one ARG, virulence factor, or plasmid replicon.

It was possible to identify 11,433 antimicrobial resistance genes, 3426 virulence factors, and 320 plasmids across the 63 analyzed genomes. To characterize the spatial distribution of antimicrobial resistance genes (ARGs), virulence factors (VFs), and plasmids in relation to temperate phage regions, genomic features were quantified across three mutually exclusive compartments: (i) within predicted temperate phage boundaries, (ii) within the immediate flanking regions (±1 kb), (iii) across the background of the host genome (all positions outside phage boundaries and flanks). This framework allows a comparison between phage-associated and genome-wide gene distributions. The distribution of major ARGs, VFs, and plasmids identified inside phages and flanking regions is shown in Figure 4A. Even with low counts, two plasmid replicon types, pO111_1 and IncY, were preferentially detected within temperate phage boundaries (27% and 20%, respectively). In addition, cationic antimicrobial peptide resistance genes were detected within phages (3% inside and 1% in flanking regions), as well as virulence factors related to adhesion (2%) and multidrug resistance genes detected inside phage regions (2%). Considering all temperate phages, 9.3% carried at least one ARG, whereas 1.4% carried virulence factors and 0.9% carried plasmid replicons. A higher prevalence was observed when restricting the analysis to medium- and high-quality temperate phages, with 22.1% carrying at least one ARG, 3.5% carrying virulence factors, and 2.3% carrying plasmid replicons, as shown in Figure 4B. However, permutation-based enrichment analyses did not identify significant positional enrichment for ARGs, virulence factors, or plasmid markers within temperate phage regions in the HQ/MQ subset.

#### 3.2.2. Integrated Defense Ecology Across Genomes

**Figure 5:**
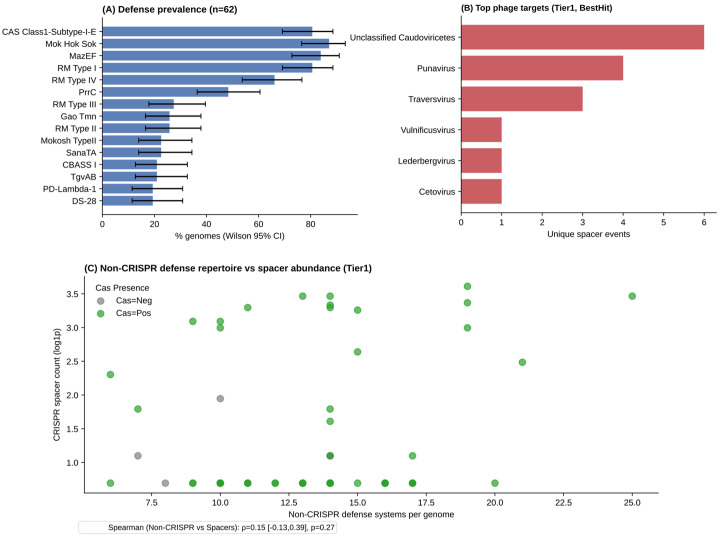
Integrated defense ecology and phage targeting across genomes. (**A**) Prevalence of the most frequent defense systems across genomes. (**B**) Most frequent phage target genera inferred from CRISPR spacer matches. (**C**) Relationship between non-CRISPR defense repertoire size and CRISPR spacer abundance per genome.

To integrate innate and adaptive antiviral defenses, we combined DefenseFinder predictions (defense system repertoire), CRISPR array summaries (total spacer counts), and spacer–target matches from sequence-based screening. Across the analyzed genomes, a total of 181 genes associated with Restriction–Modification systems, 94 MazEF toxin–antitoxin genes, 78 Mok/Hok/Sok genes, 54 CRISPR–Cas genes, and 38 PrrC-associated genes were identified, as shown in Figure 5A. At the subtype level, CRISPR–Cas subtype I-E and RM Type I were the most abundant, highlighting the predominance of innate defense mechanisms in the genomic defense landscape. Spacer hits were filtered using strict biological criteria, including minimum identity and coverage thresholds as implemented in SpacePHARER, and only phage-associated matches were retained to obtain a conservative taxonomic assignment. Taxonomic assignments of spacer targets were subsequently inferred by mapping SpacePHARER hits to NCBI nucleotide accessions, followed by lineage retrieval via the NCBI Entrez taxonomy database. Among the most frequently targeted phage genera, it was possible to identify adaptive memory against phages from the genera Unclassified Caudoviricetes (6), *Punavirus* (4), *Traversvirus* (3), *Vulnificusvirus* (1), *Lederbergvirus* (1) and *Cetovirus* (1), as presented in Figure 5B. The relationship between defense system diversity and CRISPR spacer abundance at the genome level was assessed using Spearman correlation, showing no significant correlation between them as presented in Figure 5C.

## 4. Discussion

Understanding the ecological structure of temperate bacteriophage communities is essential for elucidating phage–host interactions and dynamics of mobile genetic elements within bacterial populations. Among the *E. coli* genomes, we observed a moderate but highly consistent diversity of temperate phage genera, with a median richness of three genera per genome based on incidence data. In parallel, genomes presented a median of three temperate phage regions per host, indicating temperate phage diversity appeared relatively consistent across the analyzed strains. Together, these patterns suggest a structured and host-associated temperate phage community within the analyzed dataset, characterized by limited genus-level turnover and the absence of extreme heterogeneity across this bacterial collection [12,39].

At the population level, temperate phage diversity approached saturation, with 19 observed genera and an estimated asymptotic richness of 21.2 genera (Chao2), corresponding to a sampling coverage of 89.6%. The near-zero final slope of the accumulation curve and the recovery of 90% of the estimated diversity with only 31 genomes suggest that genus-level diversity approached saturation within the analyzed collection. These results support the interpretation that the temperate phage repertoire in this dataset shows recurrent taxonomic patterns and relatively low genus-level turnover, rather than supporting broader ecological generalization beyond this sampling context [40,41]. These observations should be interpreted within the scope of the present dataset, which comprises 63 femur-derived *E. coli* genomes from Brazilian broilers.

Phages associated with genetic richness and evenness may be fundamental for the ecological and epidemiological success of *E. coli* in intensive poultry production systems, facilitating rapid adaptation to environmental and therapeutic pressures. This is consistent with previous reports showing that APEC isolates are genetically diverse (26 O:H serotypes and 24 sequence types) and often contain virulence determinants shared with human ExPEC, in addition to widespread multidrug resistance, including clinically relevant cephalosporin resistance genes (e.g., blaCTX-M, blaSHV, blaCMY-2) carried on mobile plasmids such as IncK replicons [42].

At the genome level, half of our isolates contained at least one temperate phage region carrying an antimicrobial resistance gene. Although this descriptive frequency is notable, it should be interpreted cautiously because temperate phage predictions do not necessarily correspond to confirmed integrated prophages, and direct quantitative comparisons across studies are constrained by differences in phage-detection strategies and host genome completeness. Previous studies have reported prophage-associated ARGs or phage-linked resistance in other bacterial pathogens [43,44,45]. In our dataset, however, ARGs were detected within predicted temperate phage regions rather than uniformly within CheckV-supported integrated prophages. Accordingly, our results show that ARGs can occur in temperate phage regions, but they do not support a significant positional enrichment of ARGs within these regions. Previous studies have shown that prophages encoding antimicrobial resistance genes can improve host viability and may be associated with reduced prophage induction under antibiotic exposure, favoring the accumulation of defective prophage remnants within the host genome [46,47]. In contrast, virulence factors encoded by prophages have been linked to increased phage fitness, including improved infectivity and superinfection exclusion [46,47].

A large-scale chicken virome study by Chen et al. (2025), using shotgun metagenomic sequencing, reported a low prevalence of ARGs in viral genomes, below 1% of detected vOTUs [48]. In contrast, Kondo et al. (2021), based on whole-genome sequencing of bacterial isolates, identified prophage-associated ARGs in approximately 21% of bacterial isolates [43]. Consistently, our analysis showed a similar frequency of resistance genes within temperate phage regions (22.2%). In contrast, virulence factors and plasmid replicon markers were rare, occurring in only 6 (3.4%) and 4 (2.3%) regions, respectively. This discrepancy reflects differences in analytical resolution, as whole-genome sequencing provides much greater resolution of genome content than metagenomes [48]. These findings suggest that temperate phage regions may occasionally carry ARGs. However, the results do not provide strong evidence that they function as preferential reservoirs or statistically enriched hotspots for ARG localization in this dataset

In our analysis, temperate phage regions showed a significant enrichment of insertion sequences, whereas their flanking genomic neighborhoods were characterized by mobilome features, with MITEs and tRNA loci commonly observed near temperate phage boundaries. A previous comparative study showed the heterogeneity of temperate phage-associated regions and the difficulty of assigning accessory genes exclusively to phage genomes, underscoring the importance of genomic contexts when interpreting phages as carriers [49]. Genomic features such as tRNA loci and recombination regions have been previously associated with temperate phage integration and retention, providing a genomic background for the mobilomic patterns observed in this study [50]. In addition, host–phage interactions, including mechanisms such as superinfection exclusion, are related to prophage composition and persistence within bacterial genomes [51].

This pattern is consistent with previous reports showing that certain bacteriophages can carry antibiotic resistance genes or promote their persistence through transduction or by exerting selective pressure. Among bacterial defense systems, we identified CRISPR–Cas profiles that can therefore be interpreted as host-level footprints of prior phage exposure and selection. Our analyses revealed a diverse but uneven repertoire of CRISPR–Cas and non-CRISPR defense systems in our isolates (restriction–modification systems, toxin–antitoxin modules, and abortive infection systems) [52,53]. Global virome analyses by Paez-Espino et al. (2016) indicate that using CRISPR to identify host–virus associations captures only a limited set of viral populations, despite extensive spacer diversity, which suggests that CRISPR systems record past phage encounters rather than comprehensively shaping the viral community [54]. Consistent with this interpretation, our genomes showed interactions with adaptive memory against the genera *Punavirus*, *Traversvirus*, *Vulnificusvirus*, *Lederbergvirus* and *Cetovirus*, indicating phage exposure to these viral lineages. Beyond that, our dataset genomes are dominated by innate antiviral defense systems, particularly restriction–modification modules and toxin–antitoxin systems, with Mok–Hok–Sok and MazEF among the most prevalent. RM systems of multiple types (I, III and IV/V) were consistently detected, indicating strong selection for early recognition and cleavage of foreign DNA. Although CRISPR–Cas Class 1 subtype I-E was frequent, its presence alongside multiple abortive and toxin–antitoxin systems suggests that adaptive immunity operates as a complementary layer rather than the primary barrier to phage infection. This defense architecture therefore indicates a predominance of innate defense systems [55,56,57].

## 5. Limitations

This study aimed to analyze the presence of temperate phages in a dataset of *E. coli*. Among the limitations, it is important to mention that our analyses utilized only Illumina short-read whole-genome sequencing, which can limit the accurate reconstruction of repetitive or structurally complex regions. As a result, prophage boundaries and integration sites may not always be resolved with high confidence, and some prophage-associated sequences could be fragmented or missed. Therefore, the number of temperate phages and integrated prophages is likely underestimated, representing a conservative estimate. In addition, the dataset was restricted to femur-derived *E. coli* isolates obtained from Brazilian broilers and therefore may not represent the full population diversity of poultry-associated *E. coli*. Additional long-read sequencing would help refine phage detection and contextualization more accurately.

## 6. Conclusions

This study provides an overview of the temperate bacteriophages associated with APEC, revealing their ecological configurations and distribution across host genomes. Our analysis demonstrates that, although antimicrobial resistance genes are distributed across *E. coli* genomes, they are not significantly enriched within temperate phage regions, whereas virulence factors and plasmids are comparatively rare within phages. Temperate phages are located within broader mobilome landscapes, with insertion sequences significantly enriched inside phage regions and other mobilome features, such as MITEs and recombination-associated loci, more frequently detected in the surrounding genomic neighborhoods. Finally, the defense architecture is dominated by innate antiviral systems, particularly restriction–modification and toxin–antitoxin modules, with CRISPR–Cas systems acting as a complementary adaptive layer that records phage exposure to the host community. Overall, this study reinforces the importance of whole-genome sequencing–based analyses to identify temperate phages and to interpret their ecological configurations and associated genetic content within bacterial hosts.

## Data Availability

The bacterial genome sequencing and assembly data analyzed in this study are publicly available in the NCBI database under BioProject accession number PRJNA917297. The complete list of genome accession numbers used in the present analyses is provided in Supplementary Table S1.

## Supplementary Materials

The following supporting information can be downloaded at: https://www.mdpi.com/article/10.3390/ani16081159/s1. Figure S1: Taxonomy of all bacteriophages; Table S1: The complete list of genome accession numbers used in the present analyses.

## Abbreviations

The following abbreviations are used in this manuscript:

APEC	Avian pathogenic *Escherichia coli*
ARG	Antimicrobial resistance genes
CRISPR	Clustered Regularly Interspaced Short Palindromic Repeats
HQ	High-quality phage
MQ	Medium quality
MGE	Mobile genetic element
MITE	Miniature Inverted-repeat Transposable Element
VF	Virulence factor
VFDB	Virulence Factors Database
Phage	Bacteriophage
