# Supplementary figures and images for: Genome Characterization of Temperate Bacteriophages and Associated Genetic Features in Avian Pathogenic Escherichia coli from Brazilian Poultry
Source: Animals (Basel). 2026 Apr 10;16(8):1159. doi: 10.3390/ani16081159 (PMC13113432; doi:10.3390/ani16081159)

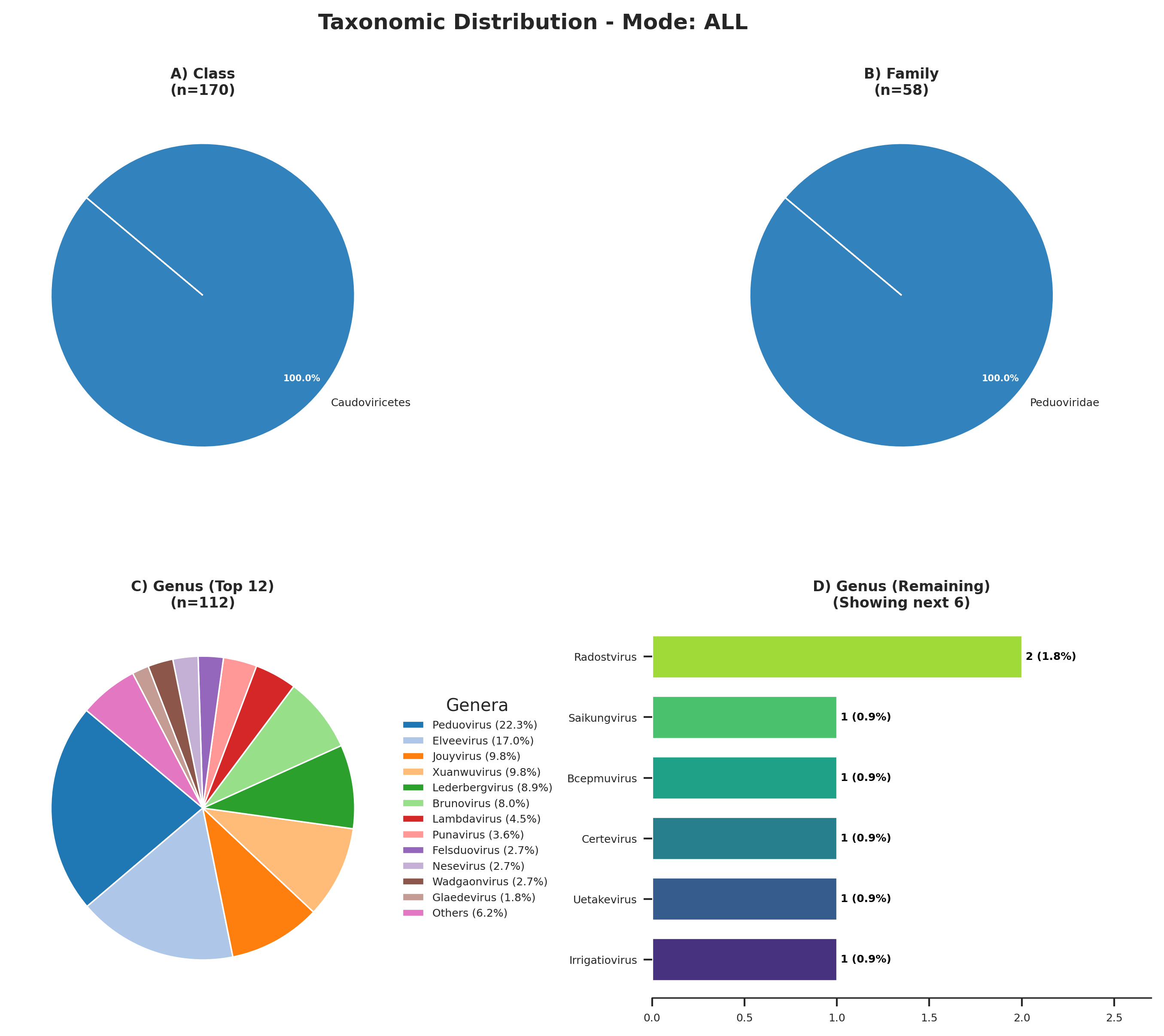

Supplement: Supplementary file 1 [file animals-16-01159-s001.zip › animals-4193159-supplementary/Figure_supplementary_1.png]
